# Effect of Elevation and Surface Roughness on Naturalness Perception of 2.5D Decor Prints

**DOI:** 10.3390/ma15093372

**Published:** 2022-05-08

**Authors:** Altynay Kadyrova, Marius Pedersen, Stephen Westland

**Affiliations:** 1 Department of Computer Science, Norwegian University of Science and Technology, 2802 Gjøvik, Norway; marius.pedersen@ntnu.no; 2 School of Design, University of Leeds, Leeds LS2 9JT, UK; s.westland@leeds.ac.uk

**Keywords:** decor, 2.5D printing, naturalness

## Abstract

Naturalness is a complex concept. It can involve a variety of attributes. In this work, we considered the effect of elevation and surface roughness on naturalness perception of 2.5D decor prints for four material categories. We found that elevation has an impact on the naturalness perception of 2.5D decor prints and that it is linked with content. The observers found lower elevation to be more natural for wood and glass 2.5D prints while there was no clear tendency for stone and metal 2.5D prints. We also found the perceptual attributes used for naturalness assessment of 2.5D decor prints. The top five ones are color, roughness, gloss, elevation, and lightness. The obtained findings can be useful for companies that produce 2.5D prints.

## 1. Introduction

Decor is one of the active interest areas in 2.5D printing based on industry feedback. Therefore, the production of 2.5D decor prints that look natural is demanded. A variety of aspects might affect the naturalness perception of 2.5D decor prints: the presence or perception of various quality attributes, illumination, and viewers’ perspectives on the quality depending on their experience and preferences, to name a few. If decor prints look natural to the viewers, then they will be considered as high quality, and consequently, will be the most demanded by the customers. As a result, it is important to investigate what parameters impact the naturalness perception of 2.5D decor prints and to what degree. In this work, we consider the effect of various quality attributes on the naturalness perception of 2.5D decor prints at a given illumination and viewing distance. To date, no study has looked specifically at 2.5D decor prints’ naturalness perception.

Elevation and naturalness were found to be in the top five most used distinct attributes during quality assessment of 2.5D prints [1]. Moreover, they are relevant from an industrial point of view as industry is investigating how elevated prints (i.e., 2.5D prints) look natural. The main feature of 2.5D prints is elevation, and it should look natural to be of high quality perceptually. The surface roughness might help to provide a realistic appearance for the prints, and it is content and material dependent. Hence, our goal is to investigate how the elevation and the surface roughness affect the naturalness perception of 2.5D decor prints. The relevance of this work is that it can provide insights on how people define the naturalness of 2.5D decor prints. Furthermore, it can be a source (or a motivation) for developing (industrial) protocols or guidelines for creating 2.5D decor prints with a natural look and finding out what level of elevation (e.g., 0.4 mm or 0.6 mm) makes a perceptually natural appearance for 2.5D decor prints. We limit to two (elevation and surface roughness) quality attributes because looking at three or more quality attributes will make the experiment long, which in turn might affect observers’ performance (i.e., leads to observer fatigue). For simplicity, by prints we mean 2.5D decor prints, by roughness we mean surface roughness, and by wood/glass/stone/metal prints we mean 2.5D prints of wood/glass/stone/metal images hereafter in the text unless specified otherwise.

This paper is organized as follows: first, we give background information about the naturalness concept (e.g., in 2D and 3D images, 2D and 2.5D prints) followed by our methodology description; afterwards, the results and discussion are given; last, we provide our conclusions and future works.

## 2. Background

We give brief background information on naturalness in images (2D, 3D) and prints (2D, 2.5D) to show that naturalness is a complex concept and combines various quality attributes.

Naturalness can be defined as a close matching between an image’s visual presentation and the understanding of the reality that is in memory [2], and it usually arises during overall image quality assessments [3]. During quality assessments, observers might use words such as natural, real, unnatural, and unreal (2D images [4], 2D prints [5,6]) and most of the time they are used to express image naturalness [3]. These attributes along with words such as edited, photoshopped, aged photo, and others were grouped into the naturalness category by Virtanen et al. [7] in their proposed image quality wheel.

Naturalness was stated as a preferential quality attribute of high level [3]. Generally, studying how, for example, chroma or sharpness variations impact the image naturalness perception is the typical approach of exploring naturalness [3]. Dependence of naturalness on a combination of various attributes was mentioned by Yoshida et al. [8] for tone-mapped 2D images. Yeganeh et al. [9] defined the naturalness of 2D images with two attributes’ (brightness and contrast) joint probability density function. Pedersen et al. [10] mentioned that naturalness can be related to, for example, color and lightness changes for 2D color prints.

Halonen et al. [3] stated that naturalness and interestingness need to be balanced when creating test images for visual quality assessments, and they worked with 2D prints. Fedorovskaya et al. [11] found that naturalness and perceptual quality have a close relationship in the context of 2D images. More specifically, they found that an image becomes unnatural due to an increase in colorfulness, which decreases the image quality. Naturalness along with details were found to be the most important/salient perceptual attributes that describe perceptual differences of 2D images [12]. There are also works dedicated to model naturalness of 2D images [13,14,15,16]. For instance, Choi et al. [13] used the sharpness and colorfulness of images, shadow-detail reproduction, and lack of washed-out appearance factors along with memory colors for 2D image naturalness modeling. The image sharpness was represented by averaged pixel-based color difference because the authors assumed that neighboring pixels’ color difference might become larger when the sharpness is increased. They used lightness to represent the shadow detail and the washed-out appearance reproduction and chroma to represent the colorfulness. They worked with both CIECAM02 and CAM02-UCS spaces.

According to Seuntiëns [17], people tolerate image distortions when rating the naturalness of both 2D and 3D images. Additionally, naturalness was found to be among the top five most used distinct attributes during quality assessment of 2.5D prints [1].

To conclude, the complexity of naturalness increases with the increase in image/print dimensionality. To our knowledge, there is no study on naturalness of higher dimensional physical prints (i.e., 2.5D).

## 3. Methodology

**Figure 1:**
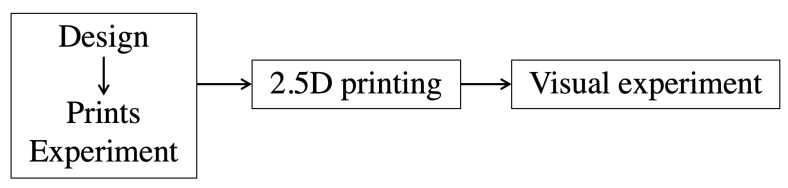
Our workflow. We started with designing the experiment and prints followed by 2.5D printing stage. Afterwards, we conducted the visual experiment.

Naturalness can be multidimensional [18] and can have various meanings depending on content. Thus, we focus on one type of content, which is decor prints. Our workflow is illustrated in Figure 1.

From the literature in the previous section, we can see that naturalness is a complex concept. In our context, realistic can mean naturalness. Naturalness was grouped together with the word real as a synonym for 2.5D prints [1]. Virtanen et al. [7] classified the word real into the naturalness category based on their data consisting of sixty-two scenes presented to the observers either via 2D print images or images on display. Thus, we define naturalness by substituting it with the term realistic representation of a print. We follow the definition of Drago et al. [12] where naturalness is considered as the extent to which an image is similar to a realistic scene. We do not refer to material properties with naturalness in this work.

### 3.1. Images

Sharan et al. [19] defined ten material categories. We worked with four material categories: wood, stone, metal, and glass. For each material category, we had a variety of content. As an example, for the wood category, we had images of wooden decor, wooden walls, and more. These four categories were selected because we believe that they represent the most used decor materials.

**Figure 2:**
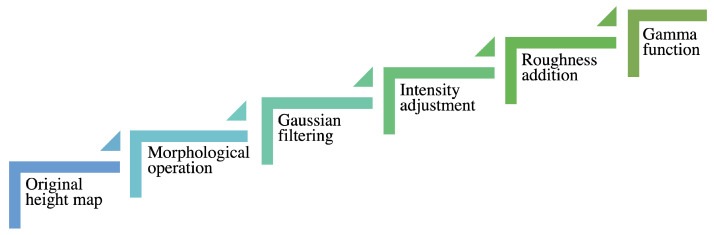
Image processing steps used for design of prints. The original height map went through several processing in the following order: morphological operation (optional, depends on image content), Gaussian filtering, intensity adjustment, roughness addition, and gamma function application.

For each material category, we had five color images, resulting in a total of 20 images. The images and their height maps (both are in 782 × 782 pixels) were reproduced from 3D textures (copyright free web site) [20] under the Creative Commons license. They contain various levels of spatial information and colorfulness [21]. The original height maps underwent the processing illustrated in Figure 2 in order to avoid printing artifacts, such as black edges due to high elevation, and to obtain visually nice prints. To reduce black edges, the height maps were processed with a Gaussian filter with a standard deviation of four, and to ensure visually nice prints, a morphological operation was applied to some images. An intensity adjustment was done to reach the intended maximum elevation. The roughness was added by direct binary search halftone blue noise with a zero mean generated by software [22] (input image was a flat grayscale at 128 with zero-mean uniform noise added, and the output image was a halftone noise image). According to Kitanovski and Pedersen [22], the direct binary search algorithm provides high-quality prints. The halftone noise image was further resized with nearest-neighbor interpolation with a resize factor of two and then cropped to the intended size. This was done to get low-frequency noise. We did not use high-frequency noise because the roughness was not visible with it during our initial tests. We applied a gamma function so that the roughness would be reproducible. We used a gamma value of 1/1.4. It was chosen to get visually nice prints via test printing of various gamma values.

We used an outdoor paper substrate. An OCE Arizona 2280GT 2.5D printer was used for the fabrication of prints. We used Alto printer mode, meaning that the elevation was opaque. The print size was 6.62 × 6.62 cm. We also added an additional 0.3 cm on each side of the substrate paper so that observers could hold the prints without touching the actual edges.

### 3.2. Elevation and Surface Roughness Levels

**Figure 3:**
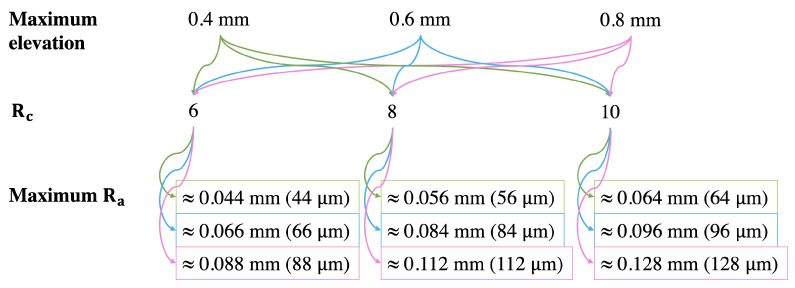
Values of maximum elevation and roughness constants (denoted as $R_{c}$) and calculated maximum roughness amount (denoted as $R_{a}$). The maximum $R_{a}$ is an approximation of what we would have physically in the prints. Three elevation levels at three roughness constants ($R_{c}$) gave nine reproductions.

The selected maximum elevation levels and roughness constants (further referred to as $R_{c}$) to make the roughness levels and the approximate maximum roughness amounts (further referred to as $R_{a}$) are presented in Figure 3. We found that prints with very low elevations look perceptually towards flat through test printing at various elevations. Moreover, it is important to consider that 2.5D prints are elevated prints. As a result, we chose the maximum elevation levels to be 0.4, 0.6, and 0.8 mm.

We used three $R_{c}$ to acquire three levels of roughness. They were multiplied with the noise image to get a height map with the roughness. These $R_{c}$ were chosen based on observations from test printing with various $R_{c}$ at various maximum elevations. With an $R_{c}<6$, the roughness looks less visible to the naked eye, especially at lower elevations. If $R_{c}>10$, the roughness does not look visually nice, especially at higher elevations. Based on these, three values of $R_{c}$ between 6 and 10 with a step of 2 were chosen.

The $R_{a}$ was calculated based on *K*-values (can be seen with a color picker in Adobe Photoshop) from the processed height maps. For example, if the *K*-values on two neighboring pixels are 100% and 89% and the maximum elevation is set to 0.4 mm, then the $R_{a}$ in that part is approximately (1−0.89)×0.4 mm =0.044 mm or 44 μm. Depending on content, the processed height maps have many pixels or few pixels with the maximum $R_{a}$. In our work, the roughness is the height difference within a local neighborhood. There were nine reproductions per image considering the three levels of elevation and roughness. This resulted in 4 categories × 5 images × 9 levels, which made a total of 180 2.5D prints for the experiment.

### 3.3. Visual Experiment

**Figure 4:**
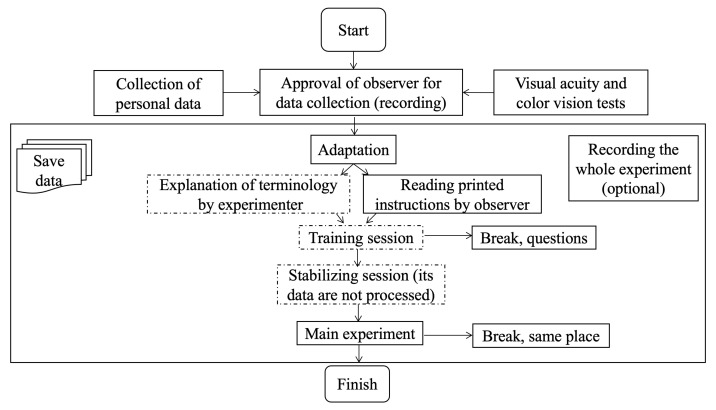
Our proposed framework for subjective quality assessment of 2.5D prints. Parts with dashed dots are optional. We included optional training and stabilizing sessions following the ITU [23] recommendation.

We did a ranking experiment because it is fast and easy for observers. Given the number of reproductions, a technique such as pair comparison would be very time-consuming. Our experimental design is illustrated in Figure 4. The observers provided their consent for participation in the experiment and for audio recording, and had a 2–3 min adaptation period to the illumination prior to starting the experiment. The 2.5D prints were presented to the observers in random order inside a light booth cabinet (Verivide CAC 60-5, illumination was 1400 lux) with D65 illumination. The prints were placed onto a 3D-printed 45° holder. As recommended by ITU [23], we did a training session so that observers could better understand the experiment’s objective and task. We used one 2.5D print (not from the total 180 prints) for a training session. After the training session, the observers also had the opportunity to ask questions before continuing. The instruction given to the observers was to rank the prints from the most to the least realistic representation of wood/stone/metal/glass decor and explain why. We did not give any physical reference to avoid observers doing fidelity matching. Instead, we provided the material category name for each print. Hence, we gave keywords as a reference. It is easier for observers to judge the realistic representation of prints when they know the material category. The distance between the prints and observers’ eyes was around 50 cm. The observers were allowed, with provided gloves, to take the prints from the holder and rotate and move them. However, as we did not consider tactility, they were not allowed to touch the prints’ surfaces. They were informed that there was no time restriction. The average duration of the experiment was 1 h and 16 min per observer. All observers finished the experiment in one session except one, who did it in two sessions.

Twenty observers (8 females and 12 males; average age: around 36 years, standard deviation: around 12 years) with normal color vision participated in the experiment, except one observer who was color deficient. Ishihara plates and a Snellen chart were used to test color vision and visual acuity, respectively. The observers were mostly Europeans, and both naive and experienced (having a background in computer science or color imaging) people were involved. It is helpful to have both naive and experienced observers to find out how they define naturalness of prints. They might assess the prints differently [24]. The experiment was run in English. The language might have impacted observers’ descriptions that they used to describe how they ranked the prints.

## 4. Results and Discussion

We analyzed the collected data both qualitatively and quantitatively to determine how people define the naturalness of 2.5D prints and what levels of elevation and surface roughness make 2.5D prints perceptually natural. We also present the limitations of our work in this section.

### 4.1. How People Define the Naturalness of 2.5D Prints?

To explore how people define the naturalness of 2.5D prints, we first present an analysis of the qualitative data. It provides perceptual attributes used by the observers as a strategy to decide on the naturalness of the 2.5D prints. Moreover, it also provides the most used perceptual attributes for examined material categories. In addition, we studied how elevation and roughness variations can affect the perception of other attributes with regard to the naturalness of 2.5D prints.

#### 4.1.1. What Are the Perceptual Attributes Linked to the Naturalness of 2.5D Prints?

The steps of qualitative data processing were: first, audio data of observers were transcribed; second, the attributes used by the observers during the experiment were extracted; third, the extracted attributes were combined into groups. We followed Virtanen et al.’s [7] approach to grouping some of the sub-attributes and in terms of visual presentation of the attributes.

**Figure 5:**
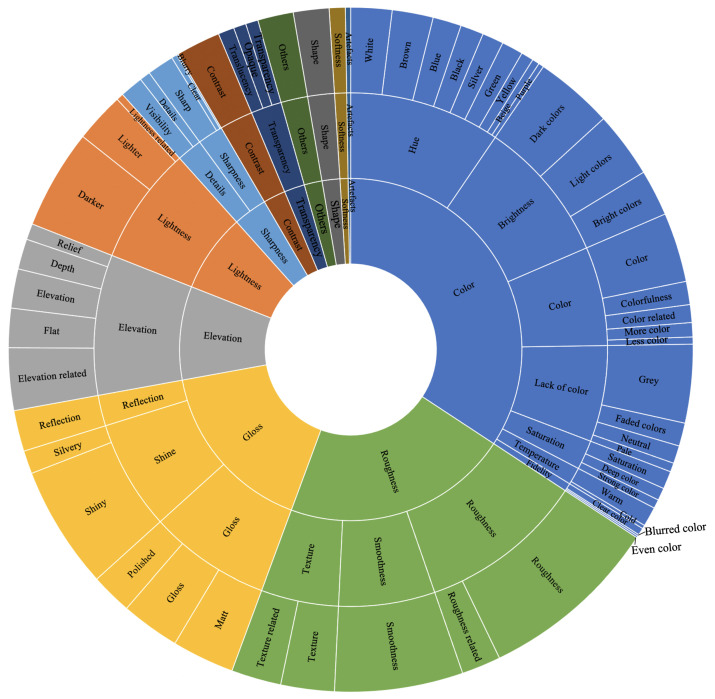
Perceptual attributes used for naturalness assessment of 2.5D prints. The most used attributes have larger areas.

Figure 5 shows the perceptual attributes used for the naturalness assessment of 2.5D prints. We defined attribute groups at three levels. In total, we found twelve level 1 attribute groups (inner circle in Figure 5). They were color, roughness, gloss, elevation, lightness, sharpness, contrast, transparency, shape, softness, artifacts, and others. For example, level 2 attribute groups of sharpness were details and sharpness. Further, level 3 attribute groups of details were visibility and details. The color-related group included chromatic color, uniform color, artificial color, and similar expressions. The texture-related group included descriptions such as even texture, visible texture, rough texture, and similar expressions whereas the roughness-related group included brown spots, noisy rough, granular, and similar expressions. The reflection group included specular reflections, diffuse specular highlights, scattering effect, and similar. The shiny group included shiny, sparkling, glittery, and similar expressions. The elevation-related group included height, altitude, 2.5D, and similar expressions. The lightness-related group included lightness and dynamic range. The others group included descriptions such as substrate, weight, clean, variability, rusty, old, and intuition (some observers ranked based on their intuitions and were not able to explain why they ranked in a specific way). The shape group included shape, size, width, and geometry. Noise and graininess were combined into the artifacts group whereas softness and hardness were combined into the softness group.

From Figure 5, we can see that the top five most used perceptual attributes for naturalness assessment of 2.5D prints by the observers were color, roughness, gloss, elevation, and lightness. They all were among the top seven most used distinct attributes during quality assessment of 2.5D prints [1].

#### 4.1.2. What Are the Most Used Perceptual Attributes Linked to the Naturalness of 2.5D Prints for Examined Material Categories?

**Figure 6:**
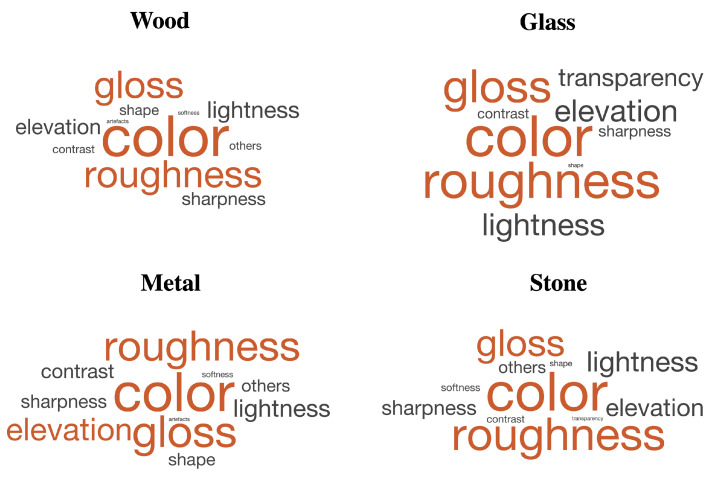
The most used level 1 perceptual attributes for wood, glass, metal, and stone material categories. The size of the attribute’s text is the frequency of its usage.

The most used level 1 perceptual attributes were identified by frequency analysis for four material categories (Figure 6). Color, roughness, and gloss were the most used perceptual attributes for four material categories. Most of the observers preferred all wood prints to be less rough, more brown, and less glossy; all glass and all metal prints to be smoother and glossier; and all stone prints to be grayer, both rougher and smoother but more towards rougher, and less glossy to be more realistic based on their explanations provided for their rankings. We can see that the transparency attribute was used only for glass prints as expected and just one time for a stone print. The observer’s criterion for that stone print was translucency in the sense that a more stone-like print should have more translucency. Artifacts were not used for stone and glass prints. Additionally, softness and attributes grouped as others were not used for glass prints.

#### 4.1.3. How Variations in Elevation and Surface Roughness Can Be Linked with the Used Perceptual Attributes for the Naturalness of 2.5D Prints?

From audio data of our observers (i.e., the explanations they provided after ranking), we can observe that various levels of elevation and roughness can impact the perception of other attributes’ presence and their variations that affect the naturalness aspect. For instance, a combination of various levels of elevation and roughness can change the color appearance and glossiness aspect. Additionally, content can impact on other attributes’ variation perception as well with regard to naturalness. We assume that higher elevation can make the print surface rougher, and similarly, lower elevation can make the print surface smoother. Moreover, we assume that more roughness can influence prints’ surfaces to appear lighter due to inter-reflections, and higher elevation can cause more contrast in prints’ surfaces. As a result, one can experience that, for example, the color of the prints varied even when color was not altered at all. Hence, we can make perception of variations of various attributes by changing just one or two attributes which in turn can impact on the naturalness perception. For 2.5D printing, it could be useful to investigate this observation in further work as it could help to produce eye-catching 2.5D products just by varying, for example, elevation levels.

Furthermore, the observers mentioned a set of factors that impact the naturalness assessment of 2.5D prints. They were grouped and named as others in Figure 5. In particular, it is worth mentioning the weight aspect that three observers mentioned. Two observers were consistent that the stone prints with more elevation should be heavier in weight, while one observer considered weight of the stone print but found the reproductions to be soft. We measured the weights of all 180 prints, and we found that the prints with more elevation had more weight than the prints with lower elevation. This is expected because there are more layers of ink in prints with more elevation and some of the observers were able to feel that.

To conclude, our finding of twelve level 1 perceptual attribute groups with which to judge the naturalness of 2.5D prints could be useful for modeling the naturalness of 2.5D prints objectively. Choi et al. [13] found, through their naturalness model, the attributes that most impact the naturalness perception of 2D images which were image sharpness and colorfulness. Thus, an objective metric for naturalness assessment of 2.5D prints can be a combination of existing models on 2D images and new models that consider the attributes found (Figure 5) in our work.

### 4.2. What Elevation and Surface Roughness Levels Make 2.5D Prints Perceptually Natural for Examined Material Categories?

In the previous subsection we found that the naturalness of 2.5D prints is linked with both elevation and roughness (Figure 5) along with other attributes, and as we changed the elevation and roughness in our prints, it is interesting to find what levels of elevation and roughness make 2.5D prints perceptually most natural. For this, we analyzed the collected data quantitatively. The raw data from the ranking experiment were converted into Z-scores. We analyzed the Z-scores image by image because, if we were to look at the combined Z-scores for all images, some effects might cancel out. For example, if one preferred a stone print to be rougher whereas another preferred a wood print to be smoother, then they would cancel out when the Z-scores for all images are combined.

**Figure 7:**
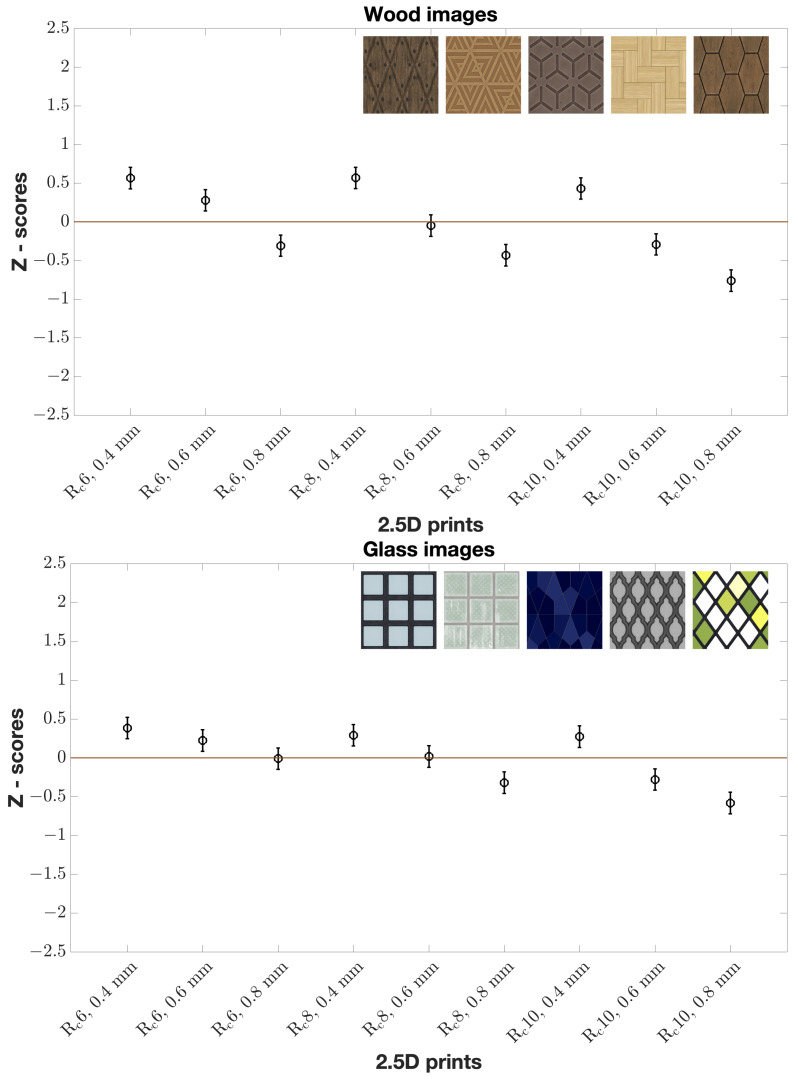
Z-scores of all images of wood and glass material categories by all observers. Mean Z-score values for nine reproductions (x-axis) are given with 95% *CI*s (represented by error bars). Z-scores have a small range. Each material category has five images.

**Figure 8:**
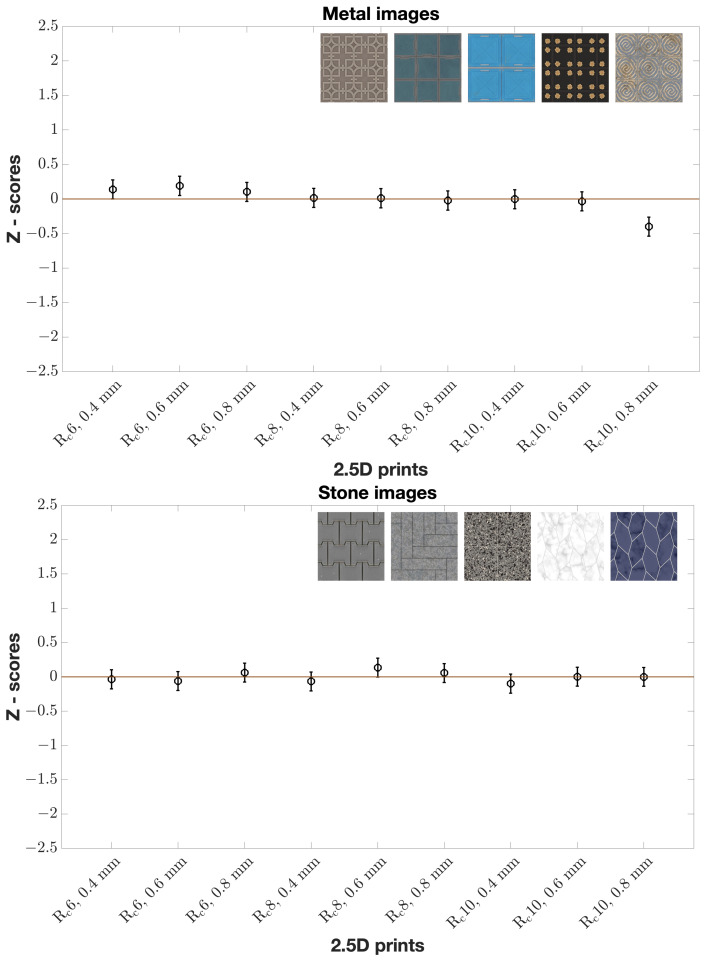
Z-scores of all images of metal and stone material categories by all observers. Mean Z-score values for nine reproductions (x-axis) are given with 95% *CI*s (represented by error bars). Z-scores have a small range. Each material category has five images.

When considering all images in each material category, we observed inverse proportionality between elevation and naturalness for all wood prints according to Z-scores (Figure 7). In other words, the observers found that wood prints should be less elevated to look natural. The same can be said of glass prints (Figure 7). No clear tendency for all stone and all metal prints (Figure 8) was found. We visualize Z-scores in error bar plots. Mean Z-scores are given by circles at the centers of the vertical lines. Confidence Interval (*CI*) was calculated as shown in Equation (1) [25].

(1) $$CI=1.96\cdot \frac{\sigma }{\sqrt{N}},$$

where *N* is the number of observations, and σ is the standard deviation which in the case of Z-score can be computed as $1/\sqrt{2}$ [26]. 95% *CI* is the mean Z-score ± *CI*. There is a statistically significant difference between the reproductions with 95% confidence, if two *CI*s do not overlap.

**Figure 9:**
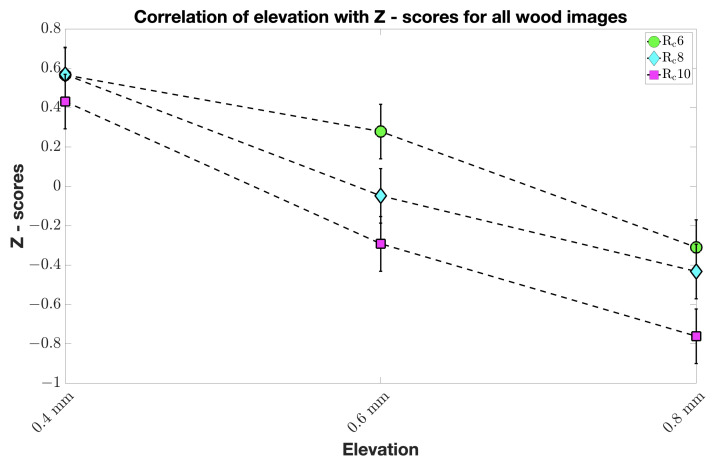
Correlation of elevation with Z-scores for all wood images. The x-axis represents 2.5D prints at various elevation levels. We can observe that the observers found lower elevation natural for all wood images, regardless of the roughness levels, as the *CI*s overlap.

We further analyzed the correlation of elevation with Z-scores and the correlation of roughness with Z-scores for all images in each material category. This showed that elevation had a correlation with Z-scores for all wood (Figure 9) and all glass images unlike all stone and all metal images. There was no clear correlation pattern of roughness with respect to the Z-scores for four material category images. It is worth mentioning that we did not find significant differences in Z-scores between naive and experienced observers and between genders.

Additionally, we used a binomial sign test on the raw data with Bonferroni correction (with a significance level of α/n, where α=0.05 is the desired alpha value and *n* is the number of comparisons: 0.05/36) [27]. Table 1 presents *p*-values obtained from the sign test for all wood images. We can observe that, at any roughness level, 0.4 mm had statistically significant difference in comparison with the other two elevation levels, and 0.6 mm had statistically significant difference in comparison with 0.8 mm. Considering both *p*-values (Table 1) and Z-scores (Figure 7), we can assume that the observers found 0.4 mm to be more natural than other two elevation levels regardless of the roughness levels for all wood prints. For all glass images (Table 2), lower roughness level resulted in a statistically significant difference compared to higher roughness levels at 0.8 mm. In other words, the observers found it more natural when all glass prints had less roughness at 0.8 mm. None of the reproductions resulted in a statistically significant difference for all stone images. The majority of reproductions resulted in no statistically significant difference for all metal images either. In addition, we looked into inter-observer variability using the Spearman correlation coefficient and found that, on average for all images, the correlation varied between observers. This shows the complexity of assessing the naturalness of 2.5D prints and the variability of the perception of overall print appearance from person to person.

To conclude, the observers preferred wood and glass 2.5D prints to have lower elevation to look perceptually natural. Furthermore, a lower elevation can make a print look smoother. In other words, we assume that the observers preferred wood and glass 2.5D prints to be less elevated and smoother.

### 4.3. Limitations

We focused on one type of content (decor) and worked with three variations of the selected quality attributes (i.e., elevation and roughness). If we were to increase the number of variations per attribute, then the experiment would have become long which would have affected observers’ performance. We sampled sparsely (i.e., 3 × 3 grid) to find an area of interest that could be investigated further as a future work. We chose one content to narrow down our scope; otherwise, it would have become difficult to differentiate the results for different contents. By focusing on one content, we generated a workflow that can be followed to study the naturalness perception of 2.5D prints in other contents. It is important to mention that the results can vary depending on the content selected. In addition, our work is useful in the selected application area—decor—which is the most active area in 2.5D printing presently.

## 5. Conclusions and Future Works

According to the literature, there have been studies where the naturalness was involved in 2D images [2,4,8,9,11,12,13,14,15,16,28], 3D images [17,29], and 2D prints [3,5,10]. Naturalness as an attribute was mentioned in Kadyrova et al.’s [1] work on attributes for the quality assessment of 2.5D prints. To our knowledge, this work is the first which studied the naturalness perception of physical 2.5D prints. Thus, our work is unique. We investigated the effect of elevation and surface roughness on the naturalness perception of 2.5D prints. We found that the observers define the naturalness of 2.5D prints with various attributes (Figure 5, Section 4.1.1). The top five attributes that the observers prefer to look at when assessing the naturalness of 2.5D prints are color, roughness, gloss, elevation, and lightness. Moreover, we found that color, roughness, and gloss are the most used attributes for four examined material categories (Section 4.1.2). Based on the results, lower the elevation, more natural the wood and glass 2.5D prints to observers (Section 4.2). We also found that the naturalness of 2.5D prints is content dependent. Thus, it is important to consider what type of content one needs to reproduce to decide on what elevation level needs to be used. Additionally, we found that a change in one or more attributes can make perception of other attributes’ variation with regard to the naturalness of 2.5D prints (Section 4.1.3).

Future work will be to explore what exact lower elevation makes 2.5D prints look perceptually natural, particularly wood prints. Additionally, it would be interesting to repeat the experiment with tactility.

## Figures and Tables

**Table 1:** *p*-Values obtained by sign test for all wood images. Green cells are those that have a statistically significant difference while red ones are those that have no statistically significant difference. Threshold used in Bonferroni correction is 0.05/36 = 0.0014.

	R6, 0.4 mm	R6, 0.6 mm	R6, 0.8 mm	R8, 0.4 mm	R8, 0.6 mm	R8, 0.8 mm	R10, 0.4 mm	R10, 0.6 mm	R10, 0.8 mm
**R6, 0.4 mm**	-	$9.6685\times 10^{-4}$	$3.7979\times 10^{-8}$	0.4839	$6.7953\times 10^{-6}$	$3.6350\times 10^{-9}$	0.2713	$3.7979\times 10^{-8}$	$3.6350\times 10^{-9}$
**R6, 0.6 mm**		-	$1.7080\times 10^{-5}$	0.0069	0.0019	$9.5837\times 10^{-7}$	0.3681	$2.6016\times 10^{-6}$	$3.6350\times 10^{-9}$
**R6, 0.8 mm**			-	$3.7979\times 10^{-8}$	0.0124	0.1936	$3.3965\times 10^{-7}$	0.4839	$6.7953\times 10^{-6}$
**R8, 0.4 mm**				-	$1.1580\times 10^{-7}$	$3.6350\times 10^{-9}$	0.1936	$3.6350\times 10^{-9}$	$2.9765\times 10^{-10}$
**R8, 0.6 mm**					-	$2.1560\times 10^{-4}$	$1.7080\times 10^{-5}$	0.0124	$1.1981\times 10^{-8}$
**R8, 0.8 mm**						-	$1.1981\times 10^{-8}$	0.2713	$9.6193\times 10^{-5}$
**R10, 0.4 mm**							-	$9.5837\times 10^{-7}$	$1.0607\times 10^{-9}$
**R10, 0.6 mm**								-	$9.5837\times 10^{-7}$
**R10, 0.8 mm**									-

**Table 2:** *p*-Values obtained by sign test for all glass images. Green cells are those that have a statistically significant difference while red ones are those that have no statistically significant difference. Threshold used in Bonferroni correction is 0.05/36 = 0.0014.

	R6, 0.4 mm	R6, 0.6 mm	R6, 0.8 mm	R8, 0.4 mm	R8, 0.6 mm	R8, 0.8 mm	R10, 0.4 mm	R10, 0.6 mm	R10, 0.8 mm
**R6, 0.4 mm**	-	0.1936	0.0019	0.4839	$2.1560\times 10^{-4}$	$9.6193\times 10^{-5}$	0.1336	$1.7080\times 10^{-5}$	$1.7080\times 10^{-5}$
**R6, 0.6 mm**		-	0.0124	0.3681	0.1336	$9.6193\times 10^{-5}$	0.7642	$4.6526\times 10^{-4}$	$6.7953\times 10^{-6}$
**R6, 0.8 mm**			-	0.0214	0.6171	$4.6526\times 10^{-4}$	0.0019	0.2713	$3.6350\times 10^{-9}$
**R8, 0.4 mm**				-	0.0124	$2.1560\times 10^{-4}$	0.3681	$2.1560\times 10^{-4}$	$4.1315\times 10^{-5}$
**R8, 0.6 mm**					-	0.0019	0.0124	0.0019	$6.7953\times 10^{-6}$
**R8, 0.8 mm**						-	$9.6193\times 10^{-5}$	0.6171	$4.6526\times 10^{-4}$
**R10, 0.4 mm**							-	$6.7953\times 10^{-6}$	$1.7080\times 10^{-5}$
**R10, 0.6 mm**								-	0.0019
**R10, 0.8 mm**									-

## Data Availability

The original images and their height maps used in this work can be found at https://3dtextures.me, accessed on 6 July 2021.
